# Plasma lipids, alcohol intake frequency and risk of Osteoarthritis: a Mendelian randomization study

**DOI:** 10.1186/s12889-023-16250-1

**Published:** 2023-07-11

**Authors:** Ming-Tao Wen, Xue-Zhen Liang, Di Luo, Jia-Cheng Li, Bo-Zhao Yan, Bo-Wen Lu, Bo Xu, Gang Li

**Affiliations:** 1 The First Clinical Medical School, Shandong University of Traditional Chinese Medicine, 250355 Shandong, China; 2 Orthopaedic Microsurgery, Affiliated Hospital of Shandong University of Traditional Chinese Medicine, 250014 Shandong, China

**Keywords:** Total cholesterol, Triglyceride, High-density lipoprotein, Low-density lipoprotein, Alcohol intake frequency, Osteoarthritis, Two-sample mendelian randomization analysis, Causal Inference

## Abstract

**Backgroud:**

Plasma lipids and alcohol intake frequency have been reported to be associated with the risk of osteoarthritis (OA). However, it remains inconclusive whether plasma lipids and alcohol intake frequency play a role in the development of OA.

**Methods:**

The study employed a comprehensive genome-wide association database to identify independent genetic loci strongly linked to plasma lipids and alcohol intake frequency, which were used as instrumental variables. The causal association between plasma lipids, alcohol intake frequency, and the risk of OA was then analyzed using two-sample Mendelian randomization methods such as inverse variance weighted (IVW), MR-Egger regression, and weighted median estimator (WME), with odds ratios (ORs) as the evaluation criteria.

**Results:**

A total of 392 SNPs were included as instrumental variables in this study, including 32 for total cholesterol (TC), 39 for triglycerides (TG), 170 for high-density lipoproteins (HDL), 60 for low-density lipoproteins (LDL), and 91 for alcohol intake frequency. Using the above two-sample Mendelian Randomization method to derive the causal association between exposure and outcome, with the IVW method as the primary analysis method and other MR analysis methods complementing IVW. The results of this study showed that four exposure factors were causally associated with the risk of OA. TC obtained a statistically significant result for IVW (OR = 1.207, 95% CI: 1.018–1.431, *P* = 0.031); TG obtained a statistically significant result for Simple mode (OR = 1.855, 95% CI: 1.107–3.109, *P* = 0.024); LDL obtained three statistically significant results for IVW, WME and Weighted mode (IVW: OR = 1.363, 95% CI: 1.043–1.781, *P* = 0.023; WME: OR = 1.583, 95% CI: 1.088–2.303, *P* = 0.016; Weighted mode: OR = 1.521, 95% CI: 1.062–2.178, *P* = 0.026). Three statistically significant results were obtained for alcohol intake frequency with IVW, WME and Weighted mode (IVW: OR = 1.326, 95% CI: 1.047–1.678, *P* = 0.019; WME: OR = 1.477, 95% CI: 1.059–2.061, *P* = 0.022; Weighted mode: OR = 1.641, 95% CI: 1.060–2.541, *P* = 0.029). TC, TG, LDL, and alcohol intake frequency were all considered as risk factors for OA. The Cochran Q test for the IVW and MR-Egger methods indicated intergenic heterogeneity in the SNPs contained in TG, HDL, LDL, and alcohol intake frequency, and the test for pleiotropy indicated a weak likelihood of pleiotropy in all causal analyses.

**Conclusions:**

The results of two-sample Mendelian randomization analysis showed that TC, TG, LDL, and alcohol intake frequency were risk factors for OA, and the risk of OA increased with their rise.

**Supplementary Information:**

The online version contains supplementary material available at 10.1186/s12889-023-16250-1.

## Introduction

Osteoarthritis (OA) is a degenerative condition that involves articular cartilage, subchondral bone, synovium, and periarticular muscles, resulting in pain, joint swelling, bone friction, and movement disorders [1–3]. It is currently the most prevalent joint disease worldwide and is frequently observed in the knee and hip joints. The incidence and prevalence of OA are positively associated with age [4, 5]. If left unaddressed, the disease can lead to muscle atrophy and osteoporosis around the joints, causing walking dysfunction and negatively impacting quality of life [6, 7]. However, modern medicine’s non-steroidal anti-inflammatory drugs (NSAIDs) carry numerous side effects, while conservative interventions such as physical therapy have limited efficacy, and late surgery and other treatments can be detrimental to patients [8]. Thus, identifying the factors that influence OA’s development and intervening early in its progression is critical.

The relationship between plasma lipids and OA has been studied by many scholars, and the consensus view is that abnormal lipids are a major risk factor for the development of OA in weight-bearing joints [9]. However, plasma lipid levels are closely related to alcohol intake, and it has been shown that excessive alcohol intake can cause abnormal lipid levels, particularly triglyceride levels, to rise significantly [10]. Excessive alcohol intake is also associated with an increased risk of developing OA [11]. Thus, plasma lipids and alcohol intake may be interrelated risk factors strongly associated with OA. In addition to the above studies, some clinical studies have also suggested that plasma lipids and alcohol intake may be risk factors for OA [11–14]. However, the perspective of these clinical studies is limited to traditional observational epidemiology, which is susceptible to confounding factors such as obesity, lifestyle and dietary patterns and reverse causation, and whether plasma lipids and alcohol intake frequency are the exact risk factors for OA remains to be investigated. To investigate the causal relationship and strength of association between OA, plasma lipid, and alcohol intake, we utilized Mendelian randomization (MR) analysis. This method was proposed by Katan in 1986 [15] and uses genetic variation as an instrumental variable to assess the causal relationship between exposure and outcome. The underlying principle is based on Mendel’s second law of inheritance, in which alleles are randomly assigned and fixed at the time of conception, similar to the random assignment of subjects to treatment and control groups in traditional RCTs. This method eliminates confounding factors and reverses causality by using the random distribution of genetic variation and simulating the randomization process of a randomized controlled experiment, avoiding interference from reverse causal associations and potential confounders encountered in traditional RCTs [16].

In this study, we employed a two-sample Mendelian randomization method to investigate the causal links between total cholesterol (TC), triglyceride (TG), high-density lipoprotein (HDL), low-density lipoprotein (LDL), alcohol intake frequency, and osteoarthritis (knee and hip osteoarthritis). Our objective was to scrutinize whether there is a genetic perspective’s causal association between plasma lipids, alcohol intake frequency, and OA risk.

## Materials and methods

### Study design

**Fig. 1:**
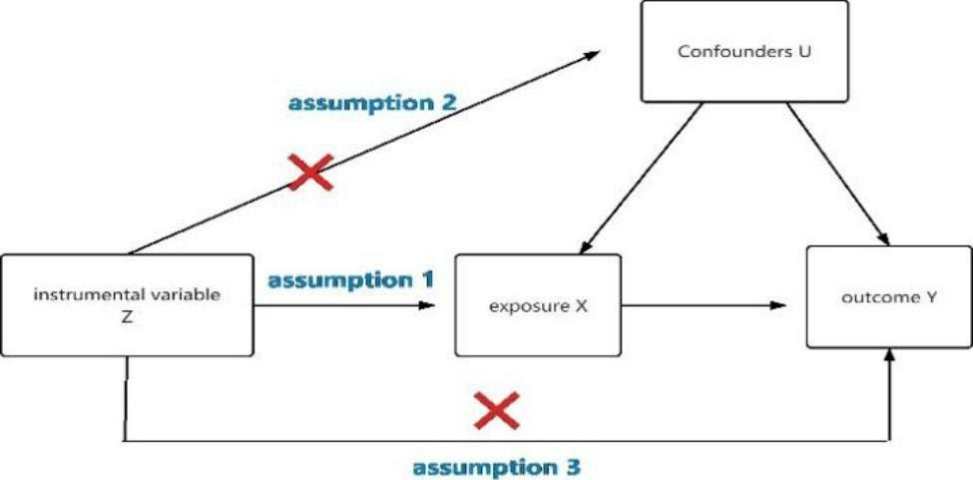
Model of the two-samples MR analysis

In this study, plasma lipids (TC, TG, HDL, LDL) and alcohol intake frequency were used as exposure factors, and single nucleotide polymorphisms (SNPs) loci significantly associated with the above exposure factors were selected as instrumental variables(IVs), and the outcome variable was OA, and the causal association analysis between exposure and outcome was performed using a two-sample MR analysis approach based on a publicly available genome wide association study (GWAS) database of large samples, and Cochran Q test to assess heterogeneity, and finally sensitivity analysis to verify the reliability of the causal association results. MR analysis needs to satisfy the following three core hypotheses: ①there is a strong association between instrumental variable Z and exposure factor X [17]; ②instrumental variable Z is not associated with any confounding factor U of the exposure-outcome association; and ③the instrumental variable Z does not affect the outcome Y, except possibly by association with the exposure X [18]. The two-sample MR study model is shown in Fig. 1.

### Data sources

The pooled data used to conduct this two-sample MR study were obtained from the IEU Open GWAS database summary website (https://gwas.mrcieu.ac.uk/). The exposure factors were TC (GWAS ID: met-d-Total_C), TG (GWAS ID: ieu-a-302), HDL (GWAS ID: ieu-b-109), LDL (GWAS ID: ieu-b-110), alcohol intake frequency (GWAS ID: ukb-b-5779), and the outcome factor was OA (GWAS ID: ieu-a-1170), and the above databases were derived from European or mixed populations. All datasets used in this study were from the public domain, and summary information is presented in Table 1.

**Table 1:** Summary of the GWAS included in this two-sample MR study

Variable	ID	Sample size	Number of SNPs	Consortium	Population	Sex	Year
TC	met-d-Total_C	115,078	12,321,875	--	European	Males and Females	2020
TG	ieu-a-302	177,861	2,439,433	GLGC	Mixed	Males and Females	2013
HDL	ieu-b-109	403,943	12,321,875	UK Biobank	European	Males and Females	2020
LDL	ieu-b-110	440,546	12,321,875	UK Biobank	European	Males and Females	2020
alcohol intake frequency	ukb-b-5779	462,346	9,851,867	MRC-IEU	European	Males and Females	2018
OA	ieu-a-1170	14,507	1,279,483	arcOGEN	European	Males and Females	2012

### Selection of instrumental variables

SNPs with significant correlation with plasma lipids and alcohol intake frequency (*P* < 5. 0 × 10^− 8^ ) were screened, and the interference of linkage disequilibrium (LD) was excluded [19], setting parameter r^2^ = 0. 001, kb = 10,000, and the echo SNPs were excluded. If the number of SNPs filtered according to the above criteria is large, each SNP should be queried on the PhenoScanner website (http://www.phenoscanner.medschl.cam.ac.uk/), SNPs affected by confounding factors that violated Mendelian randomization core hypothesis②and③were excluded, and finally valid SNPs significantly associated with exposure factors that met Mendelian core hypothesis were obtained as IVs. *F* > 10 indicates the absence of weak instrumental variable bias. If we choose publicly available GWAS databases for research, the large sample sizes typically eliminate the presence of weak instrumental variables, and therefore, there is no need to calculate the F value. However, if we utilize non-public and relatively smaller clinical databases for MR analysis, it becomes necessary to calculate the F value. The *F*-value is calculated as follows: $F = \frac{{N - k - 1}}{k} \times \frac{{{R^2}}}{{1 - {R^2}}}$, where N is the sample size of the exposed database, k is the number of SNPs, and R^2^ is the proportion of variance explained by SNPs in the exposed database. R^2^ is calculated as ${R^2} = \frac{{2 \times EAF \times (1 - EAF) \times {\beta ^2}}}{{S{D^2}}}$, where EAF is the effect allele frequency, β is the allele effect value, and SD is the standard deviation.

### Statistical analysis for mendelian randomization

We used the TwoSampleMR package (version 0.5.6) in R program (version 4.2.1) to integrate and analyze the data. In this study, inverse variance weighted (IVW) [20] was used as the main analysis method, while MR-Egger regression [21], weighted median estimator (WME) [22], simple mode and weighted mode [23] were used together for MR analysis. The principle of IVW is to weight the inverse of the variance of each IV as the weight while ensuring that all IVs are valid, the regression does not consider the intercept term, and the final result is the weighted average of the effect values of all IVs. The major difference between MR-Egger regression and IVW is that the regression takes into account the presence of the intercept term, and in addition, it also uses the inverse of the ending variance as a weight for the fit. The WME is defined as the median of the weighted empirical density function of the ratio estimates, which allows consistent estimation of causality if at least half of the valid instruments are present in the analysis.

### Heterogeneity and sensitivity test

There may be heterogeneity in the 2-sample MR analysis due to differences in analysis platforms, experimental conditions, including populations and SNPs, which may bias the estimation of causal effects. Therefore, the main IVW and MR-Egger methods were tested for heterogeneity in this study. The heterogeneity test was used to test the differences between individual IVs, and Cochran’s Q statistic and P-value were used to determine whether there was heterogeneity, and *P* < 0.1 represented the presence of heterogeneity; Pleiotropy test mainly tests the presence of horizontal pleiotropy for multiple IVs [24], and the P-value of the pleiotropy test was used in this study to measure whether there was pleiotropy in the analysis, if P > 0.05, it is considered that the possibility of pleiotropy in the causal analysis is weak; Leave-one-out sensitivity test is mainly to calculate the MR results of the remaining IVs after eliminating them one by one [25], if the estimated MR results of other IVs after eliminating one IV are very different from the total results, it means that the MR results are sensitive to that IV. The presence of pleiotropy in the analysis was also determined in this study using the MR-pleiotropy residual sum outlier (MR-PRESSO) [26].

## Results

### Instrumental variables

First of all, we screened SNPs with strong correlation with exposure in the corresponding GWAS database and removing the interference of linkage disequilibrium, and then we extracted the information of the above SNPs from the GWAS database corresponding to OA, we obtained 457 valid SNPs, including 34 for TC, 52 for TG, 194 for HDL, 81 for LDL, and 96 for alcohol intake frequency. Next, we removed the echo SNPs and the outlier SNPs obtained by MR-PRESSO test, for a total of 24 SNPs. Finally, we queried each SNP on the PhenoScanner website (http://www.phenoscanner.medschl.cam.ac.uk/) to exclude SNPs affected by confounding factors such as “age, inflammation, body mass index, type 2 diabetes”, which step eliminated a further 41 SNPs. We eventually obtained 392 IVs, including 32 for TC, 39 for TG, 170 for HDL, 60 for LDL, and 91 for alcohol intake frequency. The F-statistics corresponding to the single SNPs were all more than 10, indicating that the causal associations were less likely to be affected by weak instrumental variable bias. Basic information on the instrumental variables is in the *Supplementary Materials (Basic information on instrumental variables)*.

### Results of two-sample MR analysis

In the MR analysis results, if OR > 1, exposure is considered a risk factor for the outcome, and vice versa, exposure is considered a protective factor for the outcome. The results needed to be combined with a *P* value to determine whether they were statistically significant. IVW was the primary pooled analysis in this study, while WME complemented invalid IVs. The other three analyses were used to supplement the reliability of the results.

Table 2 shows that TC, TG, LDL, and alcohol intake frequency are all causally associated with OA and are considered risk factors for OA, with the risk of OA increasing with all four factors. Among these, TC obtained statistically significant result in IVW: OR = 1.207, 95% CI: 1.018–1.431, *P* = 0.031. It indicates that individuals exposed to TC have an approximately 20.7% increased probability of developing OA compared to individuals not exposed to this factor. TG obtained statistically significant result in simple mode: OR = 1.855, 95% CI: 1.107–3.109, *P* = 0.024. It indicates that individuals exposed to TG have an approximately 85.5% increased probability of developing OA compared to individuals not exposed to this factor. LDL had three statistically significant results: IVW: OR = 1.363, 95% CI: 1.043–1.781, *P* = 0.023; WME: OR = 1.583, 95% CI: 1.088–2.303, *P* = 0.016; weighted mode: OR = 1.521, 95% CI: 1.062–2.178, *P* = 0.026. Using IVW as an example, it suggests that individuals exposed to LDL have an approximately 36.3% increased probability of developing OA compared to individuals not exposed to this factor. Similarly, three statistically significant results were obtained for alcohol intake frequency: IVW: OR = 1.326, 95% CI: 1.047–1.678, *P* = 0.019; WME: OR = 1.477, 95% CI: 1.059–2.061, *P* = 0.022; weighted mode: OR = 1.641, 95% CI: 1.060–2.541, *P* = 0.029. Using IVW as an example, it suggests that individuals exposed to alcohol intake frequency have an approximately 32.6% increased probability of developing OA compared to individuals not exposed to this factor. The visualization of the analysis results is attached in the supplementary material *(MR results visualization charts).*

**Table 2:** MR estimates of associations between 5 types of exposure and OA risk

Exposure	Number of SNPs	MR methods	outcome	OR(95%CI)	*p-*value
TC	32	MR Egger	OA	1.352(0.951,1.923)	0.103
		WME		1 (0.780,1.283)	1
		IVW		1.207(1.018,1.431)	0.031
		Simple mode		1.109(0.701,1.757)	0.661
		Weighted mode		1.098(0.797,1.511)	0.571
TG	39	MR Egger		0.855(0.644,1.134)	0.284
		WME		0.917(0.724,1.161)	0.471
		IVW		1.003(0.835,1.205)	0.97
		Simple mode		1.855(1.107,3.109)	0.024
		Weighted mode		0.831(0.659,1.049)	0.127
HDL	170	MR Egger		0.935 (0.731,1.197)	0.596
		WME		1.128 (0.903,1.409)	0.29
		IVW		1.074(0.922,1.252)	0.357
		Simple mode		1.194(0.702,2.030)	0.515
		Weighted mode		1.106(0.909,1.346)	0.316
LDL	60	MR Egger		1.151(0.700,1.891)	0.582
		WME		1.583(1.088,2.303)	0.016
		IVW		1.363(1.043,1.781)	0.023
		Simple mode		1.521(0.776,2.980)	0.227
		Weighted mode		1.521(1.062,2.178)	0.026
alcohol intake frequency	91	MR Egger		1.003(0.641,1.571)	0.989
		WME		1.477(1.059,2.061)	0.022
		IVW		1.326(1.047,1.678)	0.019
		Simple mode		1.359(0.656,2.815)	0.412
		Weighted mode		1.641(1.060,2.541)	0.029

Abbreviations: CI, confidence interval

### Result of heterogeneity and sensitivity test

The Cochran Q test indicated intergenic heterogeneity among the instrumental variables used for TG, HDL, LDL, and alcohol intake frequency. The pleiotropy test showed no horizontal multiple validity of the results. Detailed results can be found in Table 3. Funnel plots demonstrated that the scatter of causal association effects was largely symmetrical, indicating that the results were not potentially biased. Furthermore, the “Leave-one-out” sensitivity analysis indicated that the results of the IVW analysis for the remaining SNPs, after eliminating each SNP in turn, were similar to the results of the analysis that included all SNPs. No SNPs were found to have a significant effect on the causal association estimates. The above results are visualised in the supplementary material at the end of the text (*MR results visualization charts*).

**Table 3:** results of Heterogeneity and sensitivity test

Exposure	outcome	MR methods	*p* of pleiotropy	*p* of Cochran Q
TC	OA		0.475	
		MR Egger		0.485
		IVW		0.51
TG			0.157	
		MR Egger		0.053
		IVW		0.038
HDL			0.163	
		MR Egger		0.006
		IVW		0.005
LDL			0.431	
		MR Egger		0.032
		IVW		0.034
alcohol intake frequency			0.156	
		MR Egger		0.104
		IVW		0.089

## Discussion

In this study, the causal relationship between plasma lipids, alcohol intake frequency and risk of OA was investigated using a two-sample MR analysis method using publicly available databases and a large-scale GWAS study. Compared to univariable MR, multivariable MR, and bidirectional MR research methods, Two-Sample MR provides a more intuitive reflection of the causal relationship between exposure and outcome, with advantages in simplicity and testing efficiency. It can offer researchers a more targeted and effective means of comparison.

Previous extensive research has identified dyslipidemia and alcohol consumption as risk factors for osteoarthritis (OA) and has investigated their mechanisms. It is currently understood that dyslipidemia can induce chronic inflammatory responses, and inflammatory factors can promote OA progression through various pathways. Elevated lipid levels can disrupt normal functioning of chondrocytes, affecting their metabolism and synthetic abilities, thereby leading to cartilage degradation. Additionally, high lipid levels may increase oxidative stress in the body, resulting in the production of reactive oxygen species and free radicals, which can cause oxidative damage to cells and adversely affect the health of joint tissues [27]. Similarly, excessive alcohol consumption can also trigger an inflammatory response in the body and disrupt calcium metabolism and balance, affecting calcium deposition in the bones and leading to osteoporosis and the occurrence of osteoarthritis. Moreover, alcohol intake can interfere with hormone balance, particularly estrogen levels. Estrogen plays a crucial role in maintaining bone density and regulating bone metabolism, and hormonal imbalances can increase the risk of developing osteoarthritis [11].

The results of our study support the existence of a causal relationship between TC, TG, LDL, alcohol intake frequency and OA. All of the above exposure factors are considered risk factors for OA. Our findings are consistent with the results of several previous studies. A clinical study by Nicoletta Bianca Tudorachi et al., which included 85 patients with knee osteoarthritis, showed that patients with knee osteoarthritis had higher levels of total cholesterol in their plasma compared to healthy individuals [28]. Maria Andersson et al. conducted a cross-sectional study to observe the association between metabolic factors and knee osteoarthritis. The results showed that elevated levels of total cholesterol, triglycerides, and low-density lipoprotein were all associated with the onset of knee osteoarthritis [14]. A clinical study from Huazhong University of Science and Technology in China quantified the cross-sectional and longitudinal effects of hyperlipidemia on knee osteoarthritis. The study found that each increase of 1 unit of triglyceride increased the prevalence and risk of clinical osteoarthritis by 9% and 5%, respectively [29]. Kendrick To et al. attempted to determine the true association between alcohol intake and OA and conducted a multi-year observational study that overturned the view that alcohol consumption can prevent OA [11]. T Liu et al. conducted a prospective study involving 2846 subjects with a follow-up period of 96 months. During the follow-up, it was determined that excessive alcohol consumption was significantly associated with the risk of OA [30]. The above findings suggest that TC, TG, LDL, and alcohol intake frequency are risk factors for OA, which is consistent with the results of our study. However, it is worth noting that the impact of plasma lipid levels on the incidence of OA usually requires clinical evaluation in combination with multiple lipid related indicators, which suggests that although lipid levels are closely related to the incidence risk of OA, the impact of different lipid components on the incidence risk of OA is different and should be analyzed specifically. This study explored the causal relationship between plasma lipids, alcohol intake frequency, and OA using two-sample Mendelian randomization, suggesting that the effect of alcohol intake and plasma lipids, especially TC, TG and LDL in lipids on the risk of developing OA is relatively conclusive.

In this study, stable and enduring genetic variations were utilized to directly observe the exposed elements and reduce the influence of various confounding factors such as social and lifestyle factors. There was less interference from reverse causality compared to retrospective studies. The randomized controlled trial design was not as large as the MR sample, which was closer to random allocation. The use of multiple instrumental variables allowed for more SNP specificity and yielded more accurate inferred results. Furthermore, our selection of instrumental variables was more detailed, comprehensive, and reliable than in previous studies. However, there are some limitations to this study. Firstly, the included samples primarily consist of individuals of European descent and mixed populations. The inclusion of non-homogeneous populations may introduce some bias to the results. Future research should aim to expand the sample size to include individuals of Asian ethnicities as well. Furthermore, the MR method used in this study is relatively limited, and future research should incorporate multiple MR methods for improvement and refinement, such as univariable MR, multivariable MR, and bidirectional MR research methods, to obtain more comprehensive and reliable causal associations. Additionally, due to constraints in time and funding, this study did not validate the causal associations through clinical research, which will be our future direction of work.

In summary, this study employed a two-sample Mendelian randomization (MR) approach to establish a causal link between plasma lipids, frequency of alcohol consumption, and the prevalence of osteoarthritis (OA). The study tentatively concluded that there is a positive causal relationship between the two factors. Although the specific mechanism remains unclear, these findings broaden our understanding of the risk factors associated with OA. The timely screening, prevention, and treatment of OA patients hold positive implications for reducing its prevalence and merit further research, demonstration, and application. Moreover, the methodology used in this study can serve as a methodological reference for investigating causal associations between other etiological factors and diseases. The utilization of Mendelian randomization methods can broaden the perspective of clinicians in researching causal relationships between etiological factors and disease outcomes. This approach is also applicable to exploring risk factors for other diseases. It is hoped that in the future, the scientific validity of this method can be further validated through clinical experiments.

## Electronic supplementary material

Below is the link to the electronic supplementary material.

Supplementary Material 1

## Data Availability

The datasets [Exposure/Outcome] for this study can be found in the [IEU Open GWAS] [https://gwas.mrcieu.ac.uk/].

## References

[1] Martel-Pelletier J, Barr AJ, Cicuttini FM, Conaghan PG, Cooper C, Goldring MB, Goldring SR, Jones G, Teichtahl AJ, Pelletier JP (2016). Osteoarthritis. Nat Rev Dis Primers.

[2] Yao Q, Wu X, Tao C, Gong W, Chen M, Qu M, Zhong Y, He T, Chen S, Xiao G (2023). Osteoarthritis: pathogenic signaling pathways and therapeutic targets. Signal Transduct Target Ther.

[3] Jiang Y (2022). Osteoarthritis year in review 2021: biology. Osteoarthritis Cartilage.

[4] Allen KD, Thoma LM, Golightly YM (2022). Epidemiology of osteoarthritis. Osteoarthritis Cartilage.

[5] Prieto-Alhambra D, Judge A, Javaid MK, Cooper C, Diez-Perez A, Arden NK (2014). Incidence and risk factors for clinically diagnosed knee, hip and hand osteoarthritis: influences of age, gender and osteoarthritis affecting other joints. Ann Rheum Dis.

[6] Favero M, Belluzzi E, Ortolan A, Lorenzin M, Oliviero F, Doria A, Scanzello CR, Ramonda R (2022). Erosive hand osteoarthritis: latest findings and outlook. Nat Rev Rheumatol.

[7] Palazzo C, Nguyen C, Lefevre-Colau MM, Rannou F, Poiraudeau S (2016). Risk factors and burden of osteoarthritis. Ann Phys Rehabil Med.

[8] Mandl LA (2019). Osteoarthritis year in review 2018: clinical. Osteoarthritis Cartilage.

[9] Sekar S, Crawford R, Xiao Y, Prasadam I (2017). Dietary Fats and Osteoarthritis: insights, Evidences, and New Horizons. J Cell Biochem.

[10] Volcik KA, Ballantyne CM, Fuchs FD, Sharrett AR, Boerwinkle E (2008). Relationship of alcohol consumption and type of alcoholic beverage consumed with plasma lipid levels: differences between Whites and African Americans of the ARIC study. Ann Epidemiol.

[11] To K, Mak C, Zhang C, Zhou Y, Filbay S, Khan W (2021). The association between alcohol consumption and osteoarthritis: a meta-analysis and meta-regression of observational studies. Rheumatol Int.

[12] Loef M, Ioan-Facsinay A, Mook-Kanamori DO, van Willems K, de Mutsert R, Kloppenburg M, Rosendaal FR (2020). The association of plasma fatty acids with hand and knee osteoarthritis: the NEO study. Osteoarthritis Cartilage.

[13] Schwager JL, Nevitt MC, Torner J, Lewis CE, Matthan NR, Wang N, Sun X, Lichtenstein AH, Felson D (2022). Association of serum low-density lipoprotein, high-density lipoprotein, and total cholesterol with development of knee osteoarthritis. Arthritis Care Res (Hoboken).

[14] Andersson M, Haglund E, Aili K, Bremander A, Bergman S (2022). Associations between metabolic factors and radiographic knee osteoarthritis in early disease - a cross-sectional study of individuals with knee pain. BMC Musculoskelet Disord.

[15] Katan MB (1986). Apolipoprotein E isoforms, serum cholesterol, and cancer. Lancet.

[16] Pierce BL, Burgess S (2013). Efficient design for mendelian randomization studies: subsample and 2-sample instrumental variable estimators. Am J Epidemiol.

[17] Emdin CA, Khera AV, Kathiresan S, Mendelian Randomization (2017). JAMA.

[18] NING L, SUN J (2023). Associations between body circumference and testosterone levels and risk of metabolic dysfunction-associated fatty liver disease: a mendelian randomization study. BMC Public Health.

[19] Hemani G, Zheng J, Elsworth B, Wade KH, Haberland V, Baird D, Laurin C, Burgess S, Bowden J, Langdon R et al. The MR-Base platform supports systematic causal inference across the human phenome. Elife 2018, 7. 10.7554/eLife.34408 PMC5976434 29846171

[20] Burgess S, Bowden J, Fall T, Ingelsson E, Thompson SG (2017). Sensitivity analyses for robust causal inference from mendelian randomization analyses with multiple genetic variants. Epidemiology.

[21] Slob EAW, Groenen PJF, Thurik AR, Rietveld CA (2017). A note on the use of Egger regression in mendelian randomization studies. Int J Epidemiol.

[22] Bowden J, Davey Smith G, Haycock PC, Burgess S (2016). Consistent estimation in mendelian randomization with some Invalid Instruments using a weighted median estimator. Genet Epidemiol.

[23] Hartwig FP, Davey Smith G, Bowden J (2017). Robust inference in summary data mendelian randomization via the zero modal pleiotropy assumption. Int J Epidemiol.

[24] Burgess S, Thompson SG (2017). Interpreting findings from mendelian randomization using the MR-Egger method. Eur J Epidemiol.

[25] Gronau QF, Wagenmakers EJ (2019). Limitations of bayesian leave-one-out Cross-Validation for Model Selection. Comput Brain Behav.

[26] Verbanck M, Chen CY, Neale B, Do R (2018). Detection of widespread horizontal pleiotropy in causal relationships inferred from mendelian randomization between complex traits and diseases. Nat Genet.

[27] Zhuo Q, Yang W, Chen J, Wang Y. Metabolic syndrome meets osteoarthritis. Nat Rev Rheumatol. 2012 Dec;8(12):729–37. 10.1038/nrrheum.2012.135 22907293

[28] Tudorachi NB, Totu T, Eva I, Bărbieru B, Totu EE, Fifere A, Pinteală T, Sîrbu PD, Ardeleanu V. Knee osteoarthritis in relation to the risk factors of the metabolic Syndrome Components and Environment of Origin. J Clin Med 2022, 11(24). 10.3390/jcm11247302 PMC9781325 36555918

[29] Zhou M, Guo Y, Wang D, Shi D, Li W, Liu Y, Yuan J, He M, Zhang X, Guo H (2017). The cross-sectional and longitudinal effect of hyperlipidemia on knee osteoarthritis: results from the Dongfeng-Tongji cohort in China. Sci Rep.

[30] Liu T, Xu C, Driban JB, McAlindon T, Eaton CB, Lu B (2022). Excessive alcohol consumption and the risk of knee osteoarthritis: a prospective study from the Osteoarthritis Initiative. Osteoarthritis Cartilage.

