# Supplementary material for: Plasma lipids, alcohol intake frequency and risk of Osteoarthritis: a Mendelian randomization study
Source: BMC Public Health. 2023 Jul 11;23:1327. doi: 10.1186/s12889-023-16250-1 (PMC10337179; doi:10.1186/s12889-023-16250-1)

MR results visualization charts

Supplementary figure 1 Scatterplot of total cholesterol and osteoarthritis risk

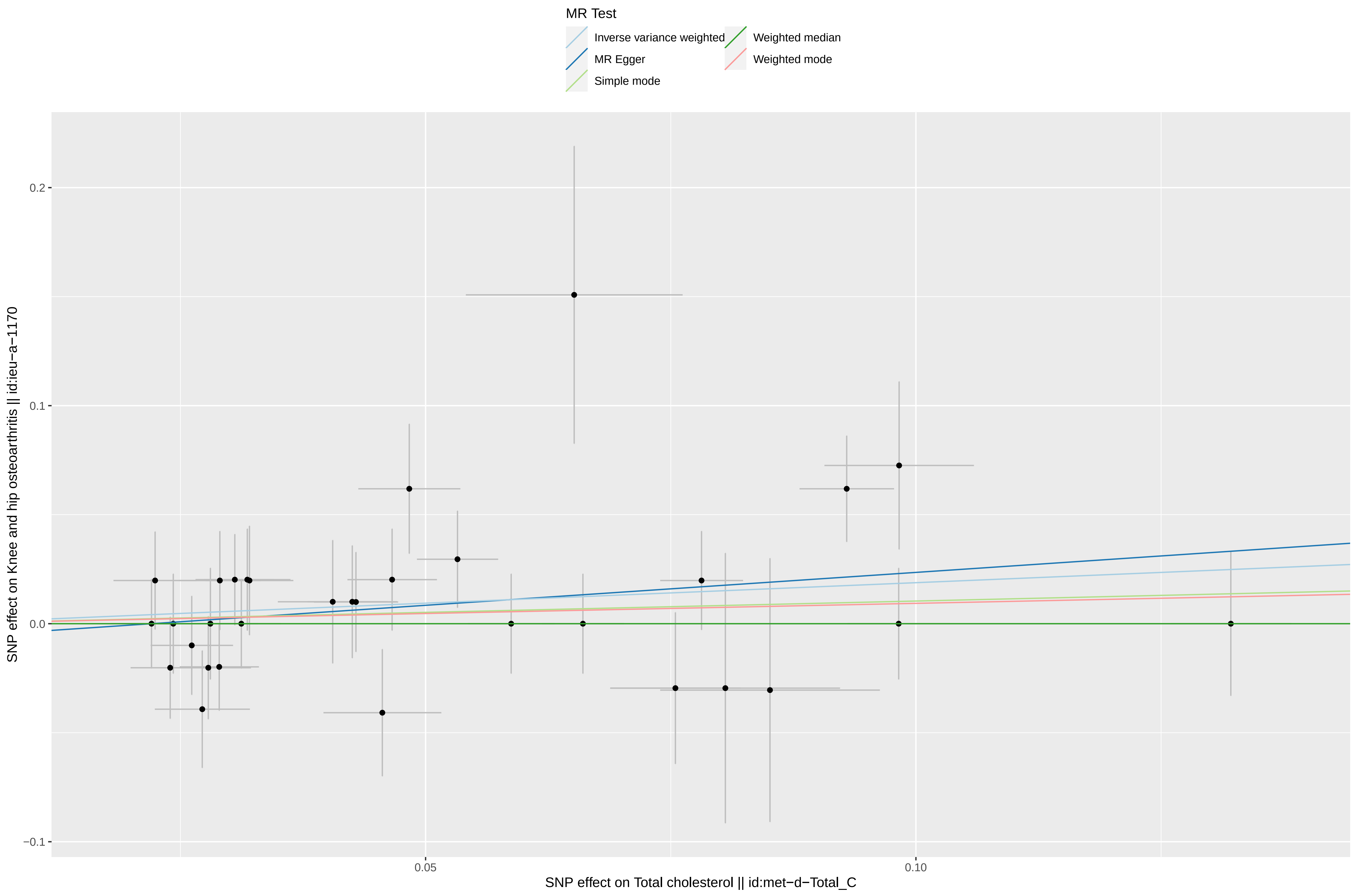

Supplementary figure 2 Funnel plot of total cholesterol and osteoarthritis risk

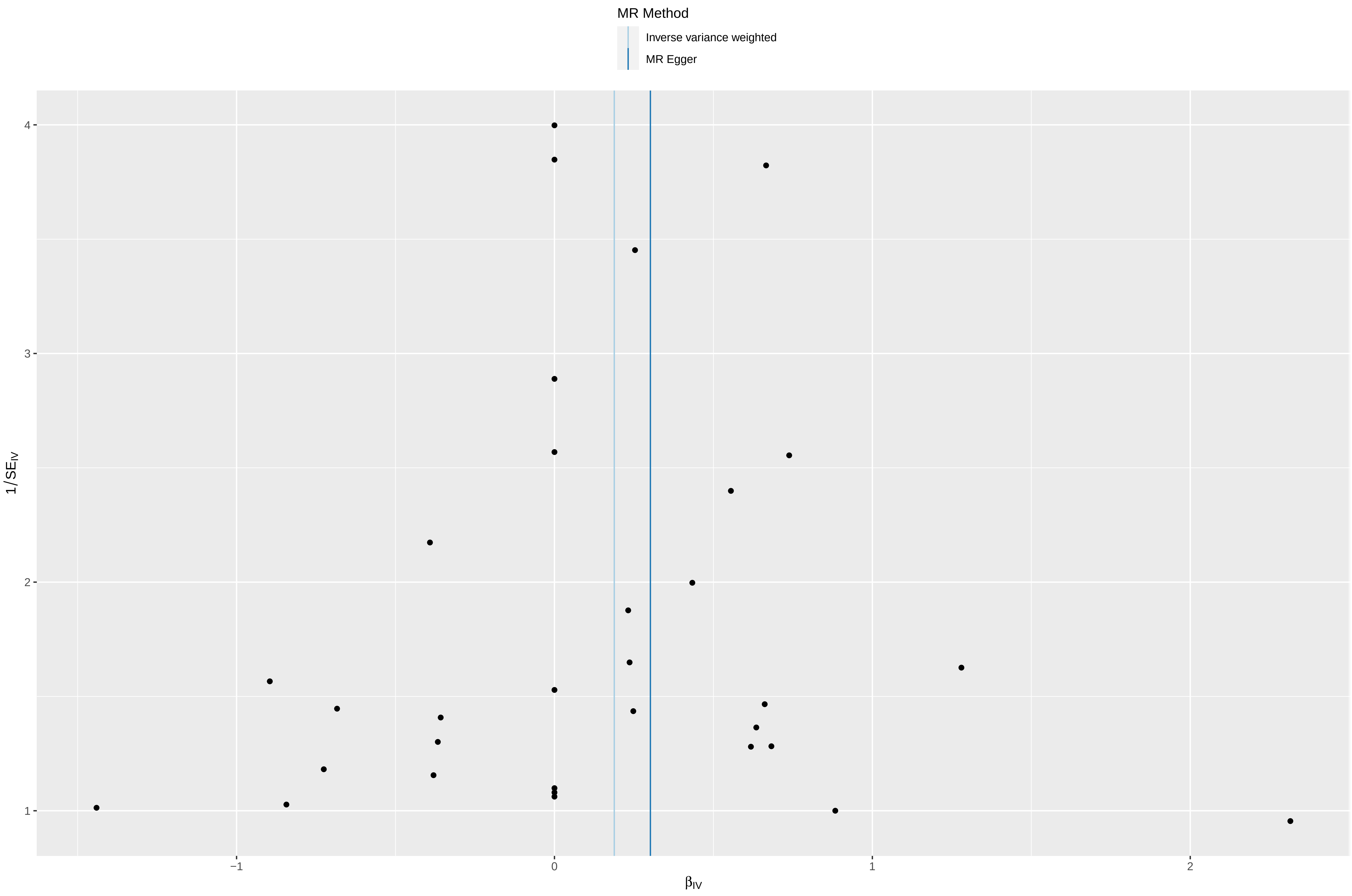

Supplementary figure 3 Forest plot of total cholesterol and osteoarthritis risk

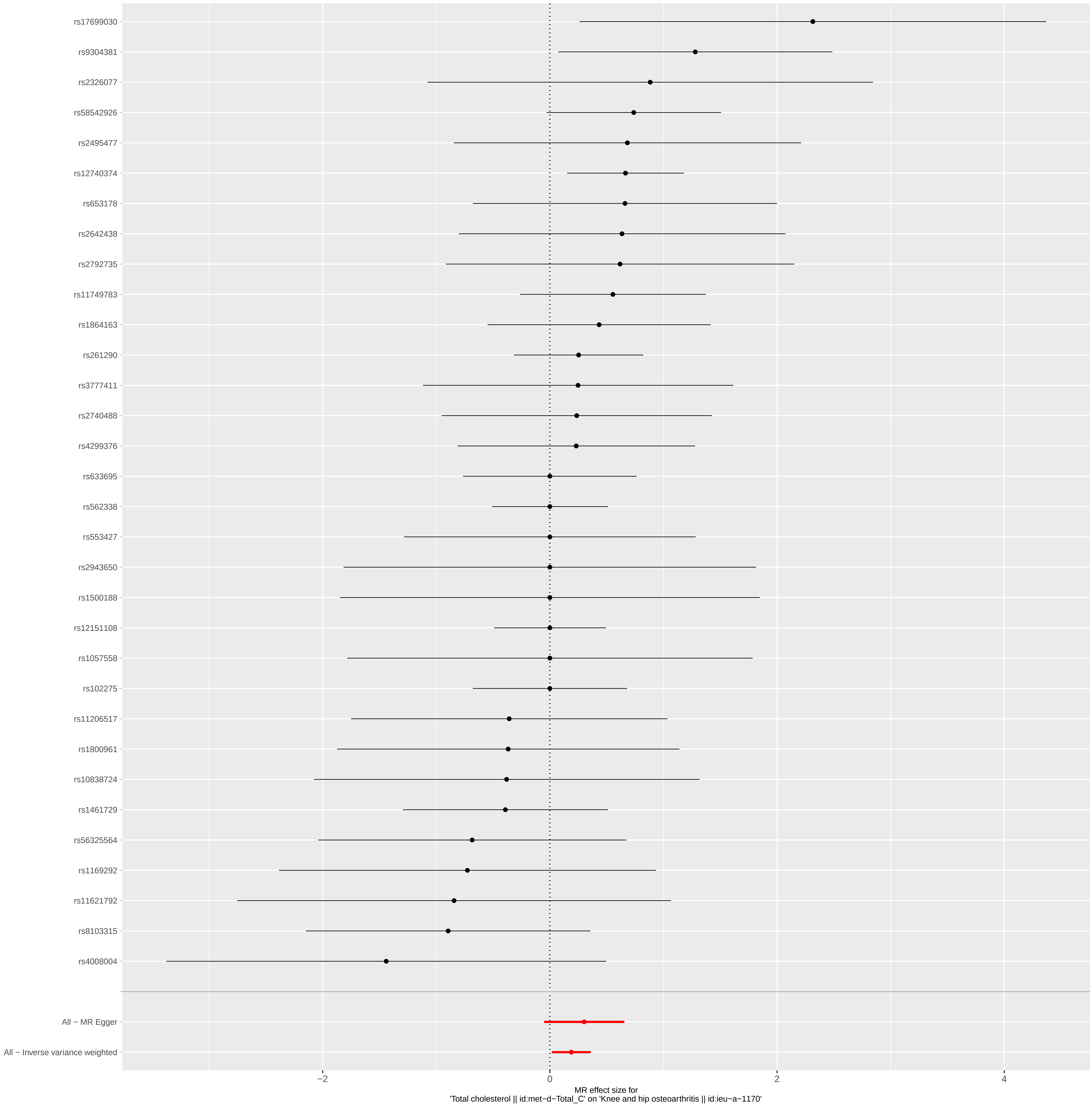

Supplementary figure 4 Sensitivity analysis of the "leave-one-out" method for total cholesterol and osteoarthritis risk

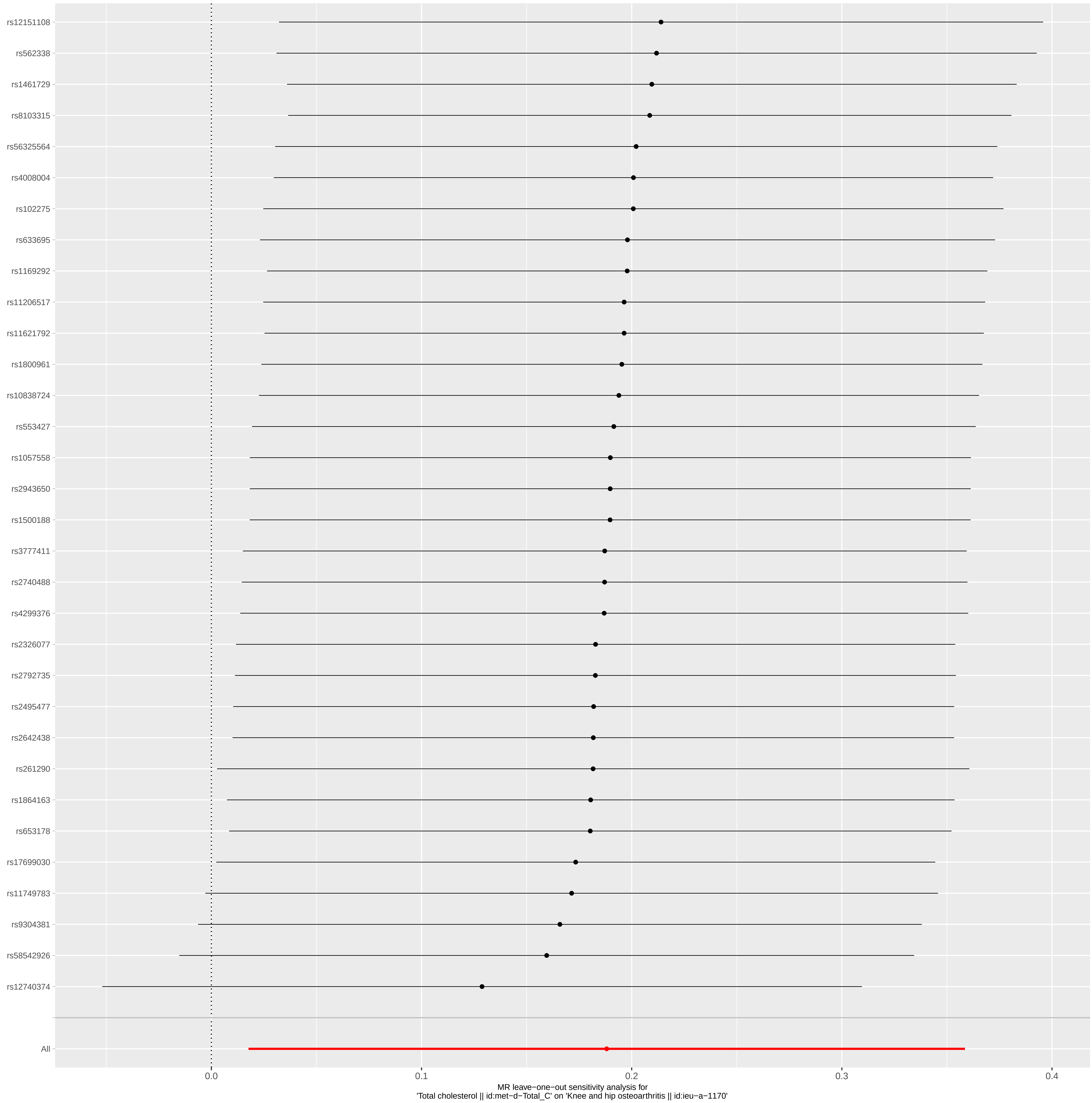

Supplementary figure 5 Scatterplot of triglyceride and osteoarthritis risk

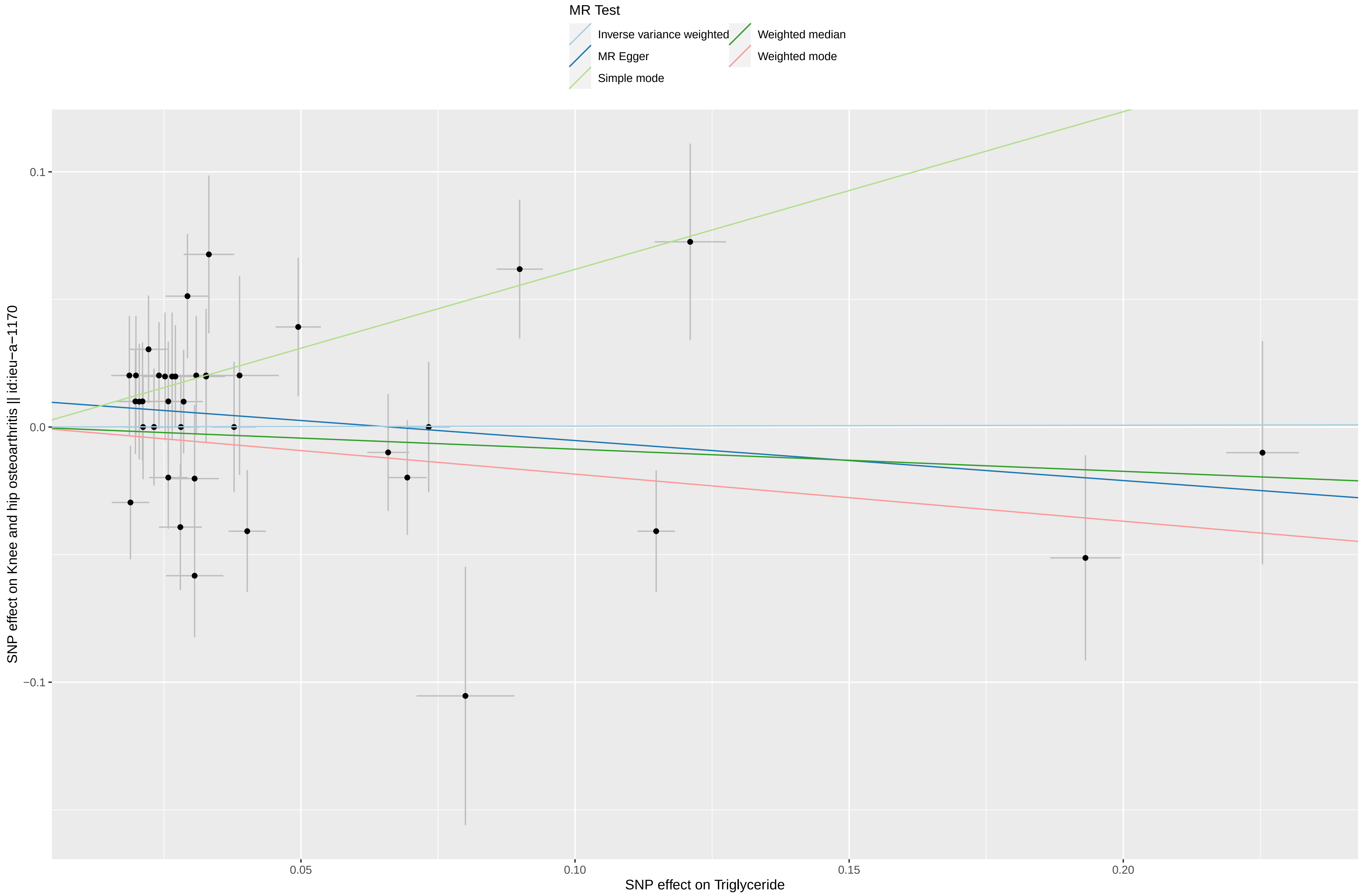

Supplementary figure 6 Funnel plot of triglyceride and osteoarthritis risk

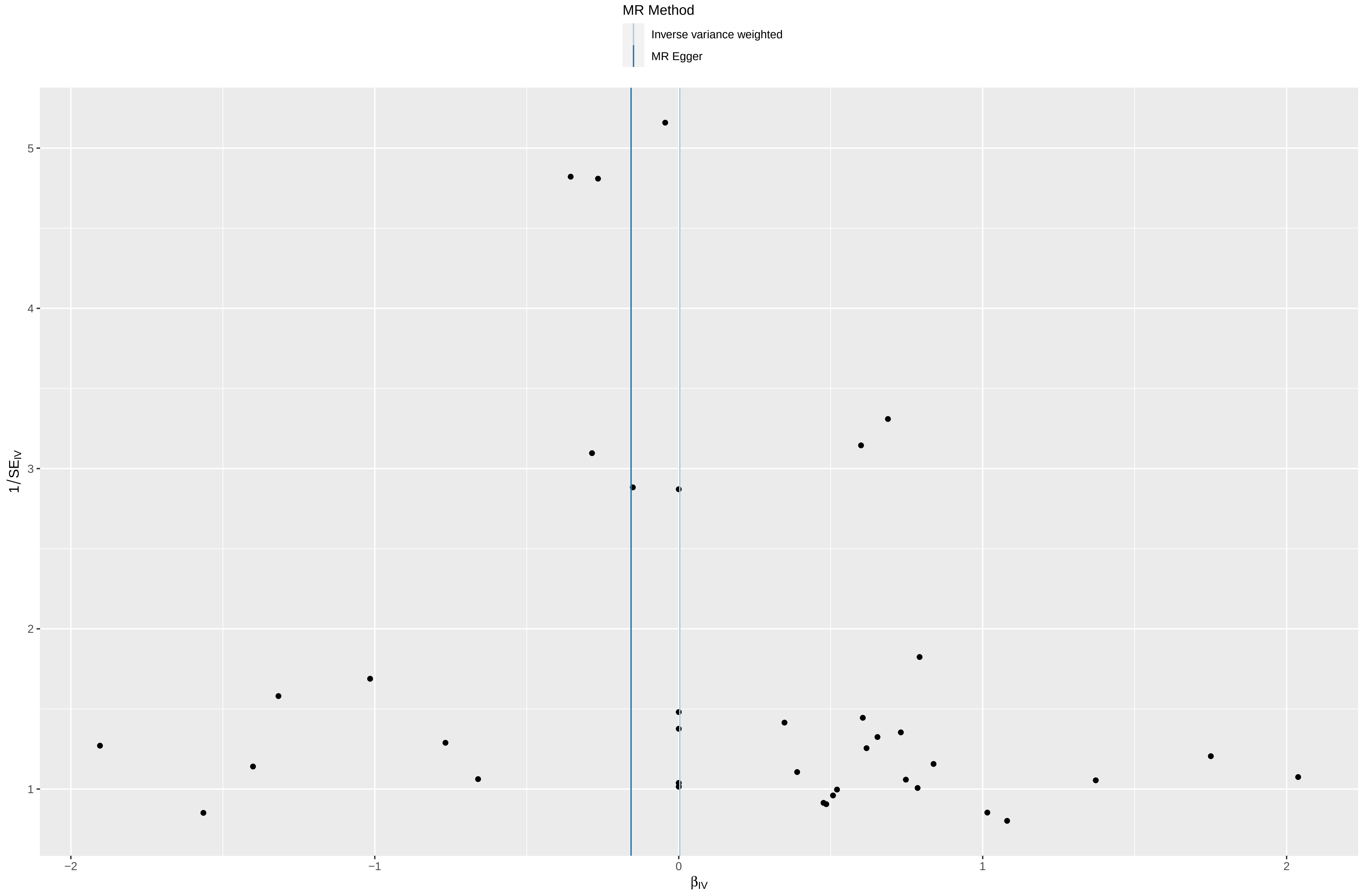

Supplementary figure 7 Forest plot of triglyceride and osteoarthritis risk

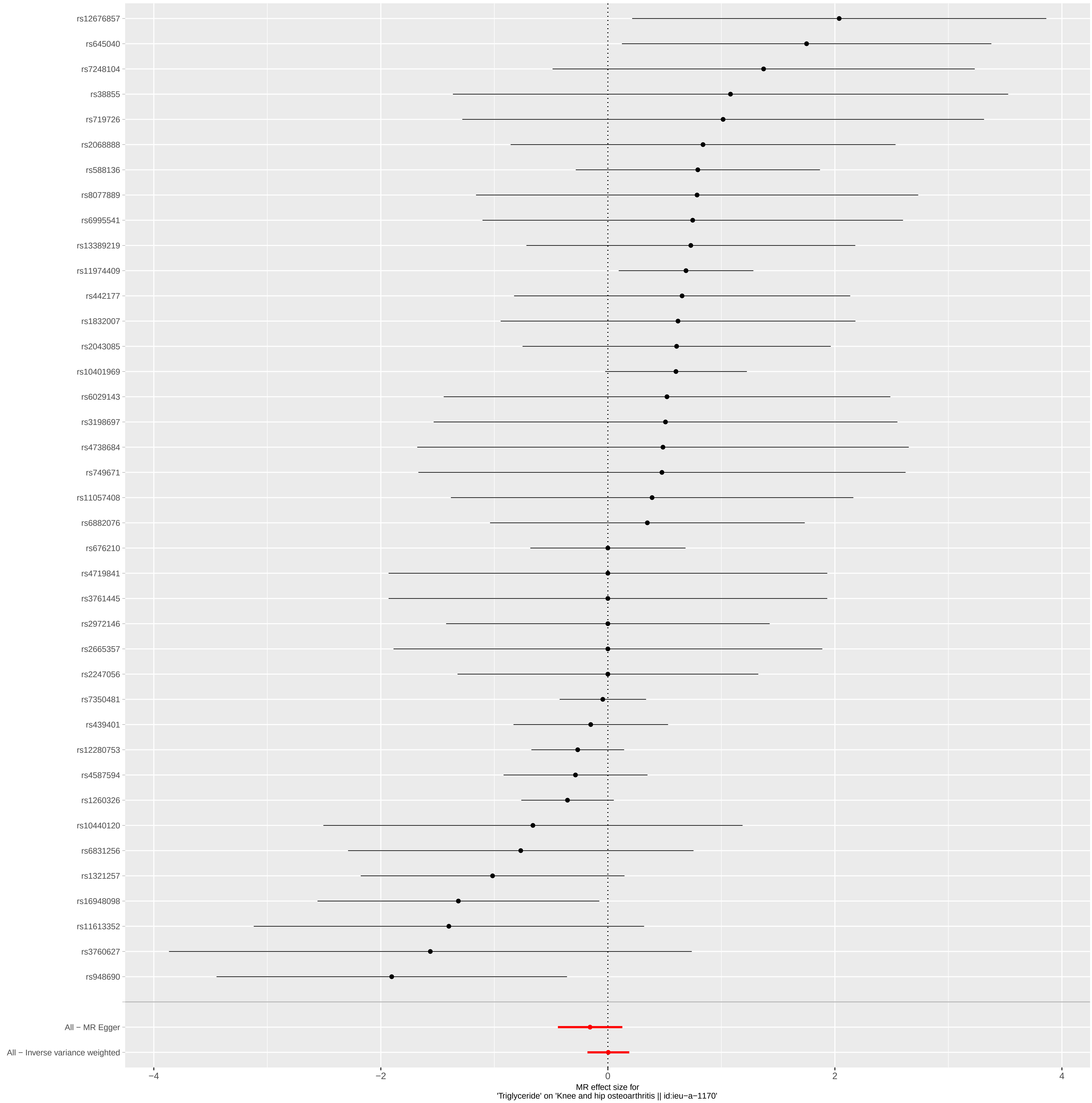

Supplementary figure 8 Sensitivity analysis of the "leave-one-out" method for triglyceride and osteoarthritis risk

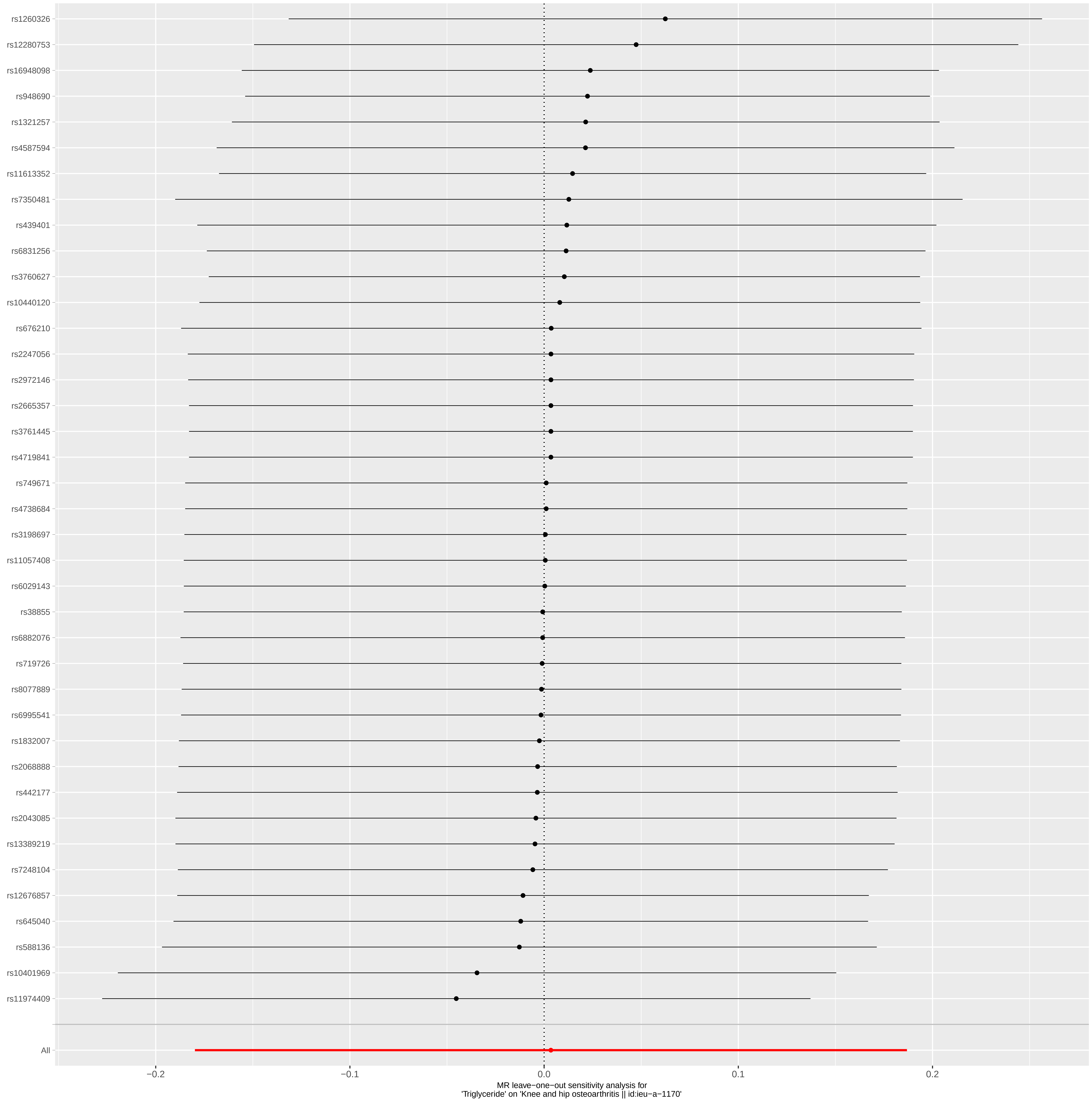

Supplementary figure 9 Scatterplot of high-density lipoprotein and osteoarthritis risk

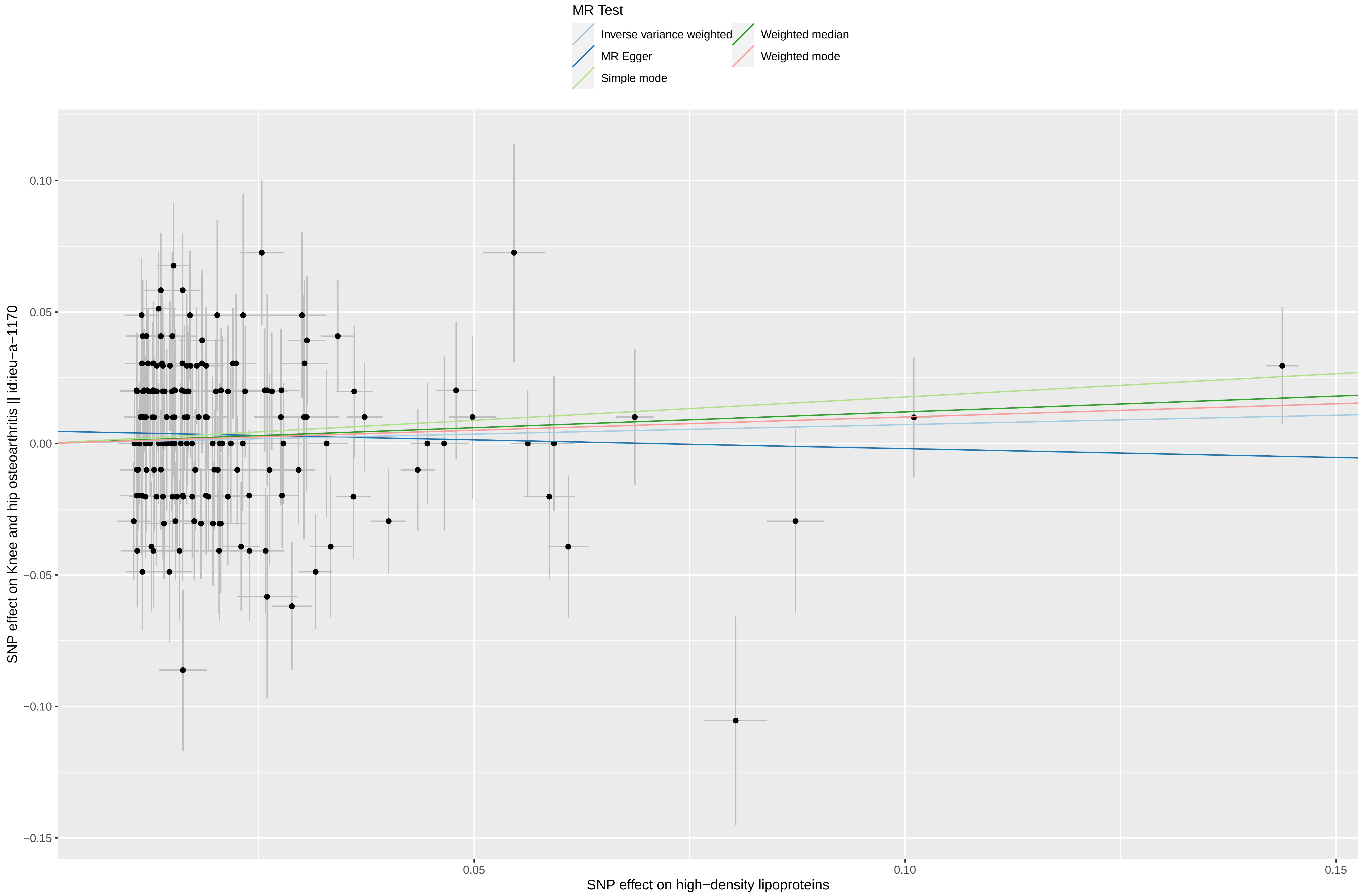

Supplementary figure 10 Funnel plot of high-density lipoprotein and osteoarthritis risk

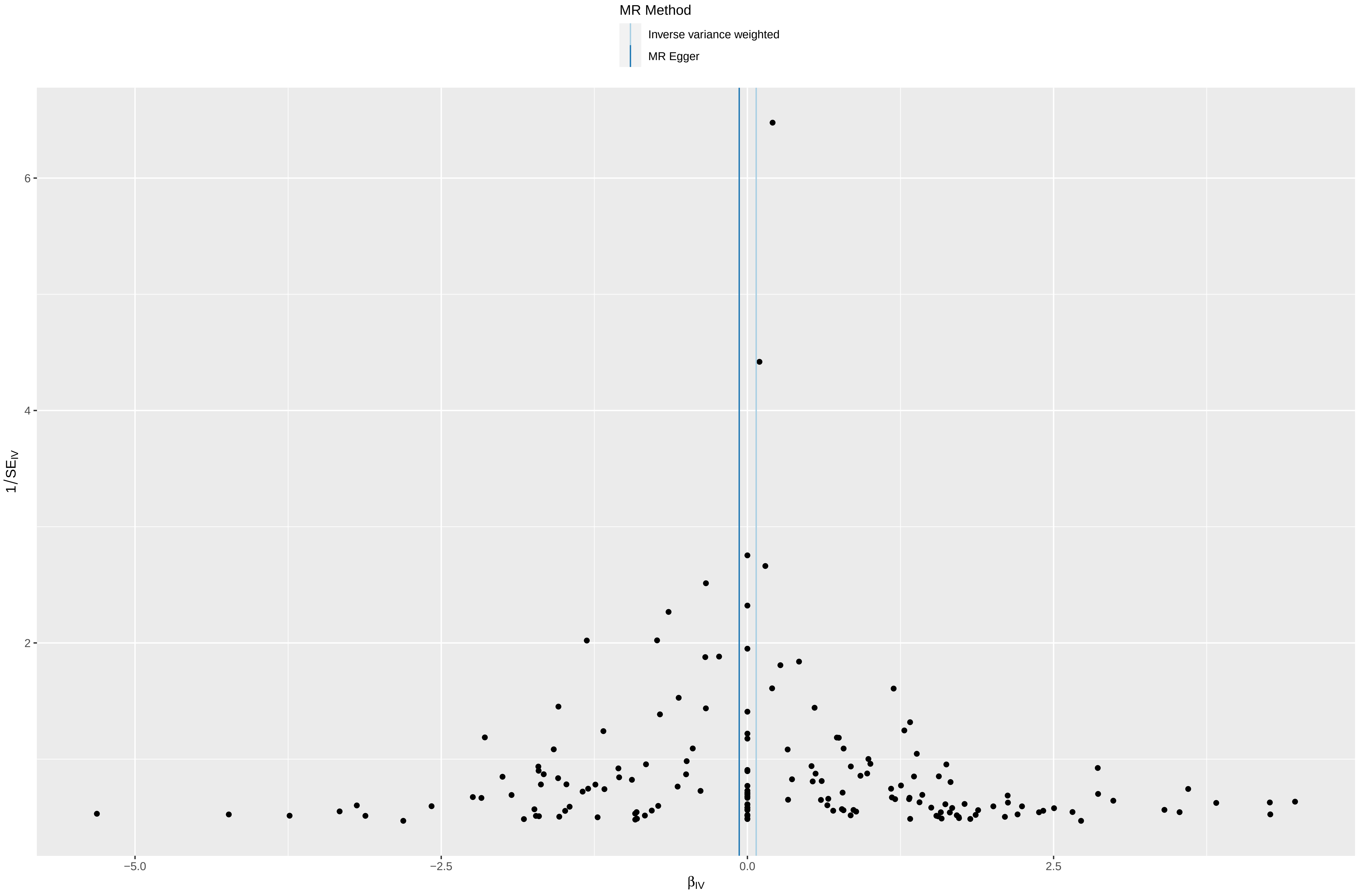

Supplementary figure 11 Forest plot of high-density lipoprotein and osteoarthritis risk

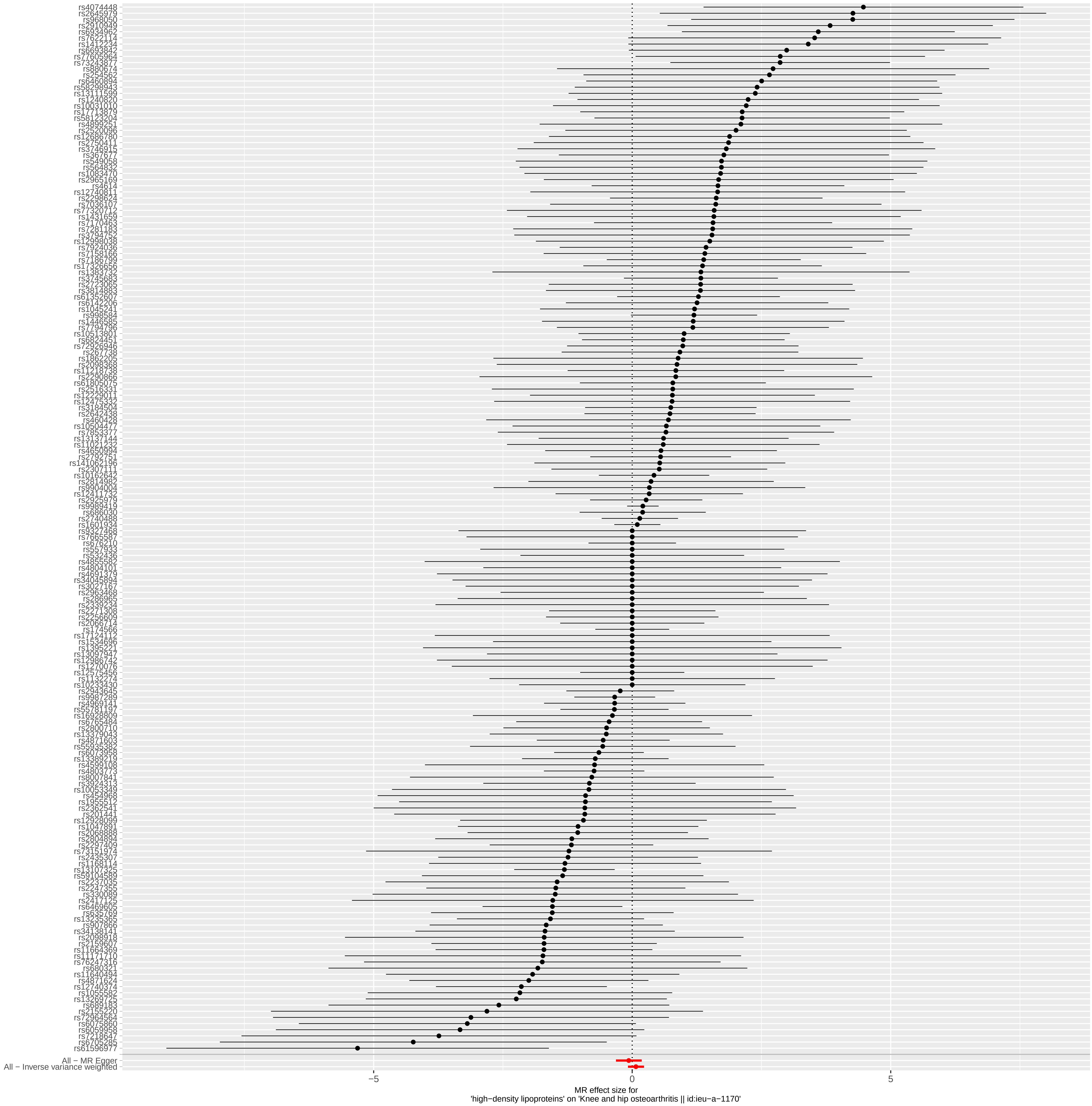

Supplementary figure 12 Sensitivity analysis of the "leave-one-out" method for high-density lipoprotein and osteoarthritis risk

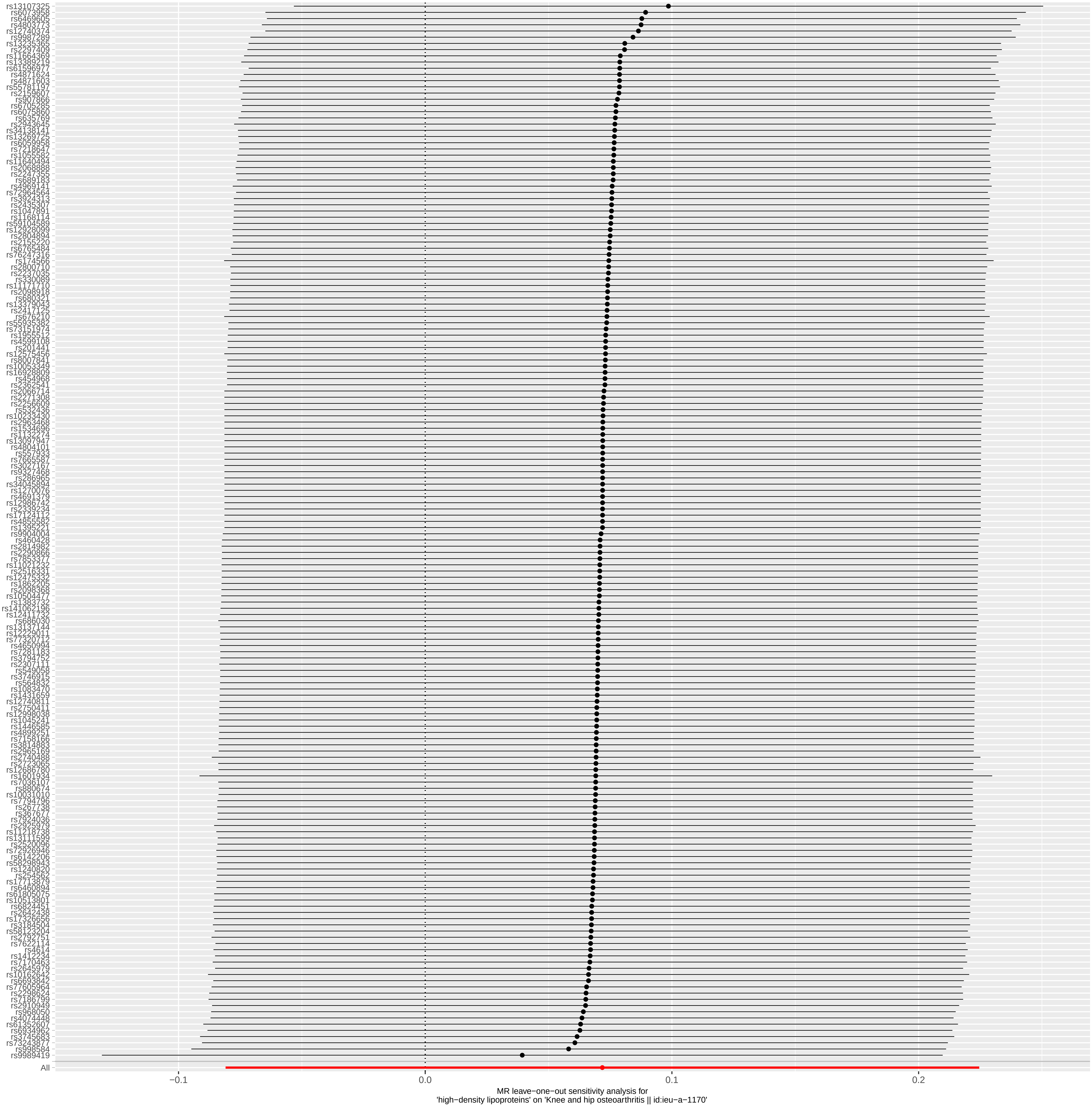

Supplementary figure 13 Scatterplot of low-density lipoprotein and osteoarthritis risk

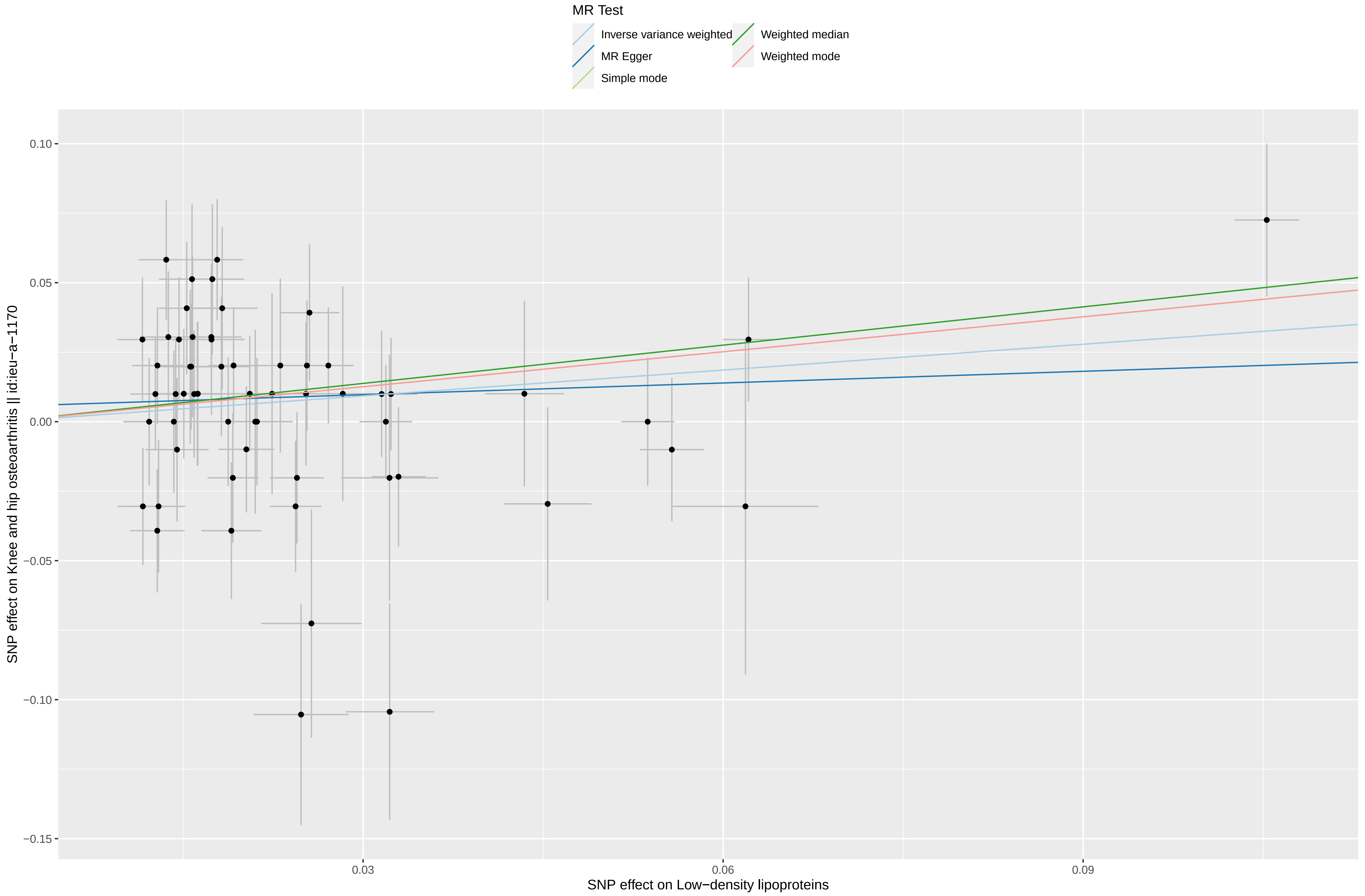

Supplementary figure 14 Funnel plot of low-density lipoprotein and osteoarthritis risk

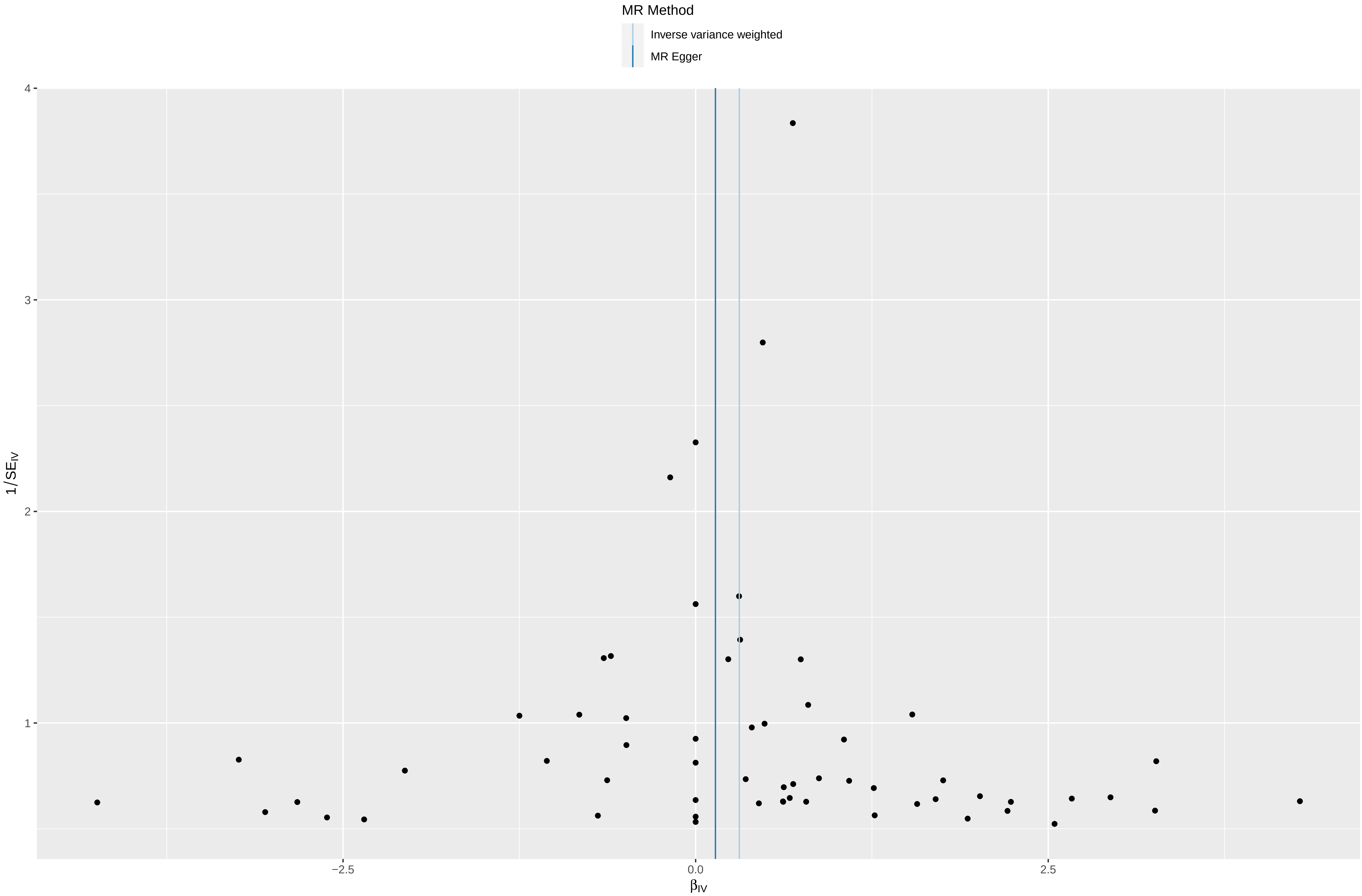

Supplementary figure 15 Forest plot of low-density lipoprotein and osteoarthritis risk

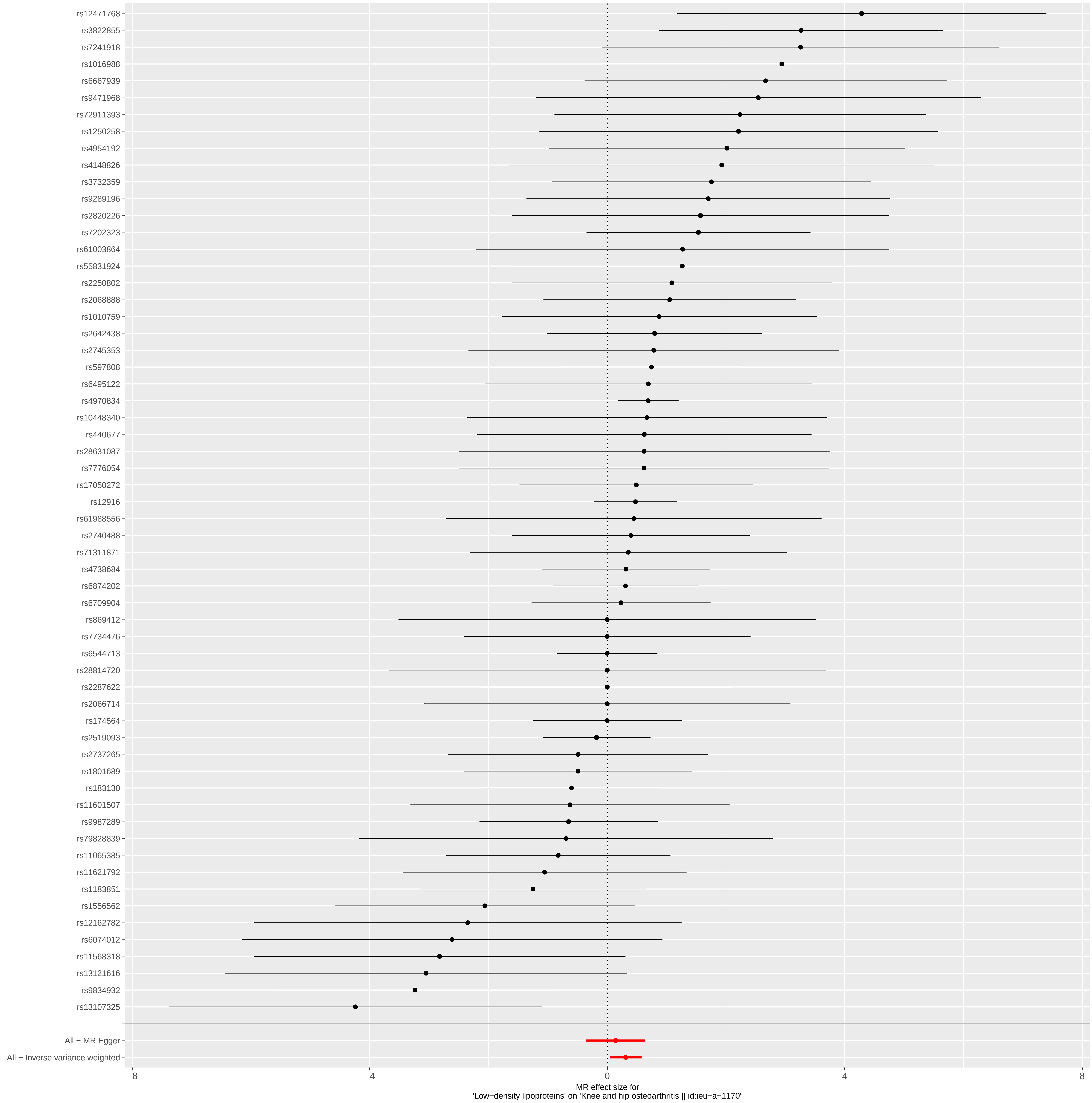

Supplementary figure 16 Sensitivity analysis of the "leave-one-out" method for low-density lipoprotein and osteoarthritis risk

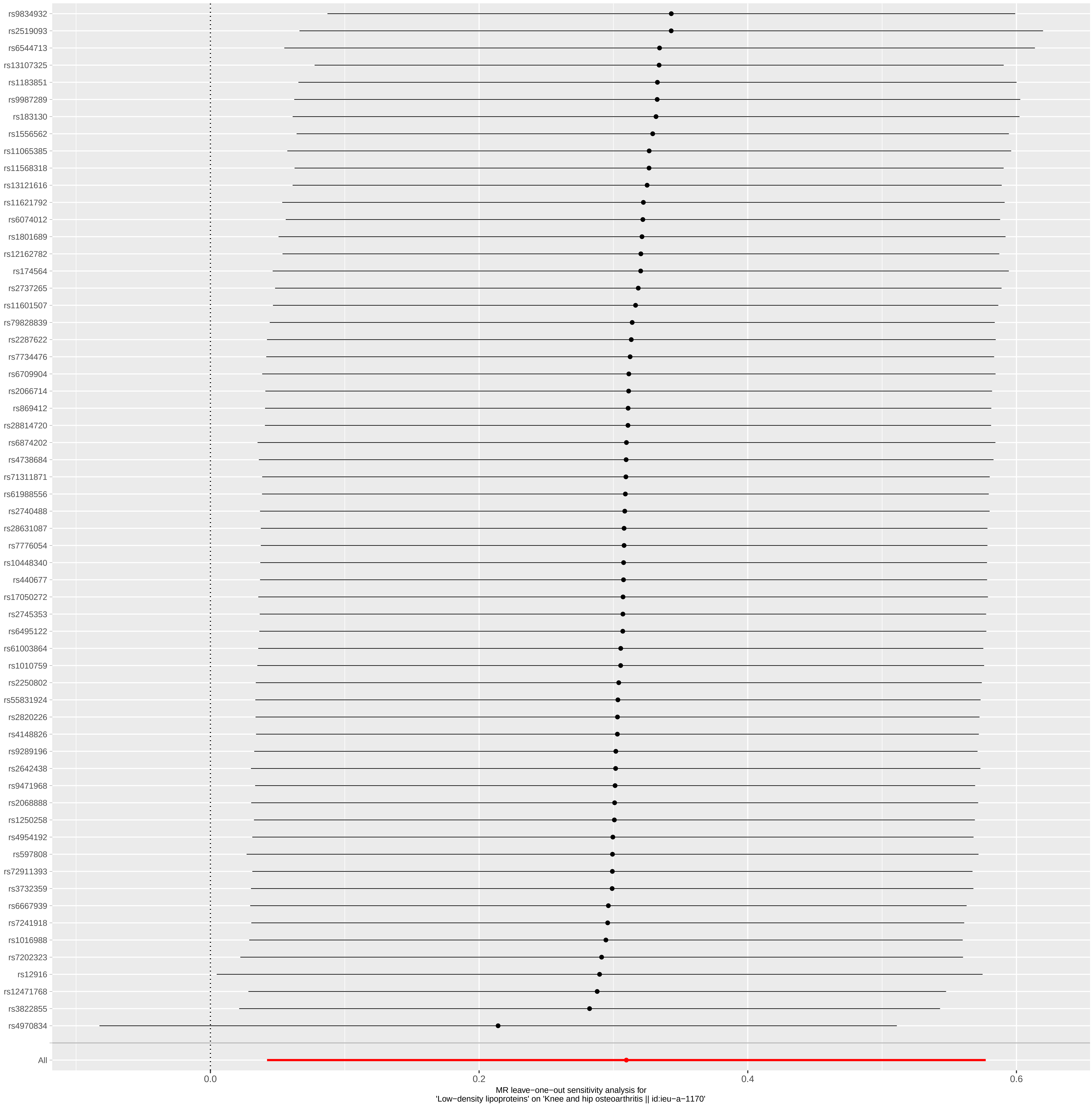

Supplementary figure 17 Scatterplot of alcohol intake frequency and osteoarthritis risk

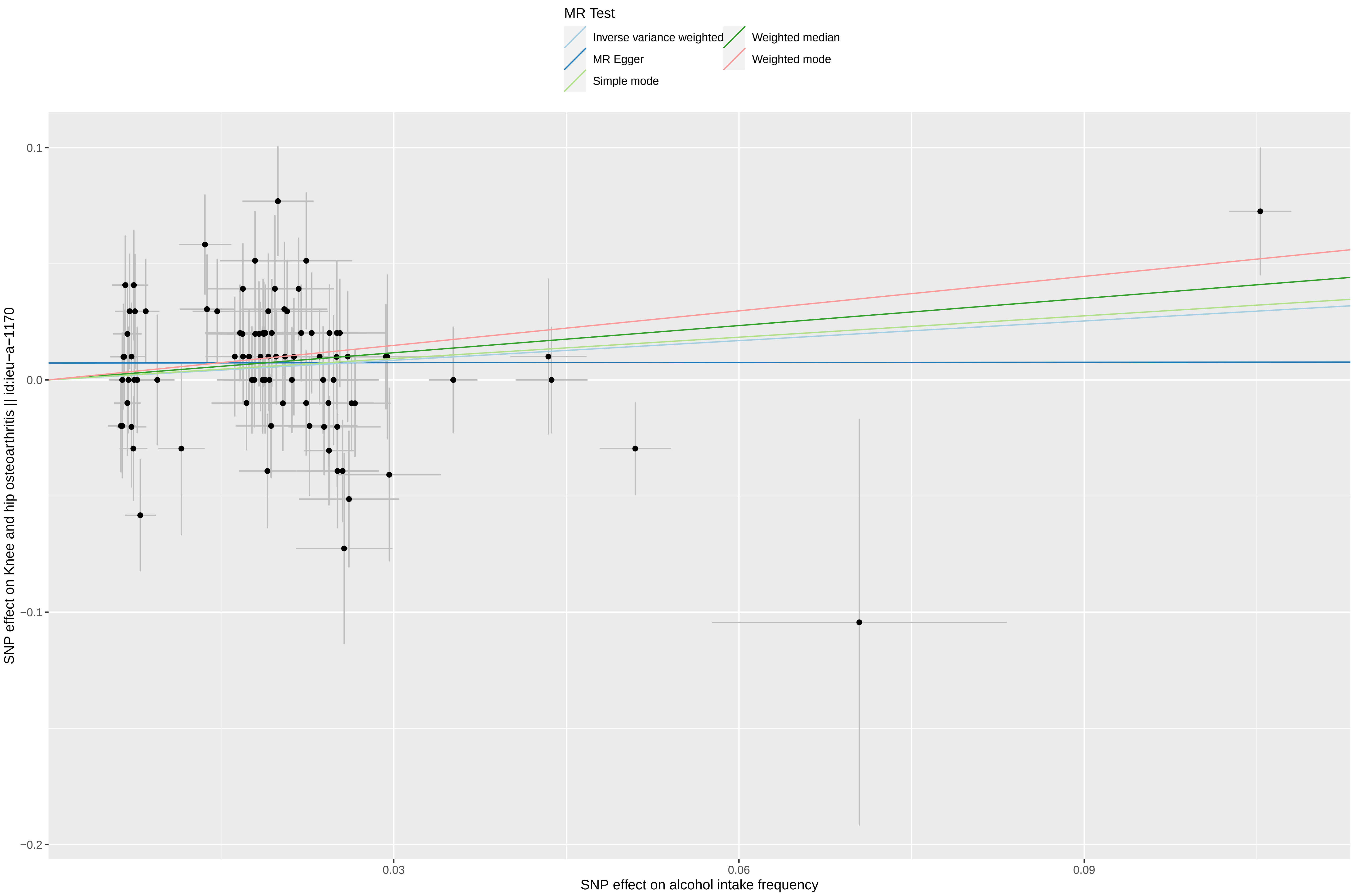

Supplementary figure 18 Funnel plot of alcohol intake frequency and osteoarthritis risk

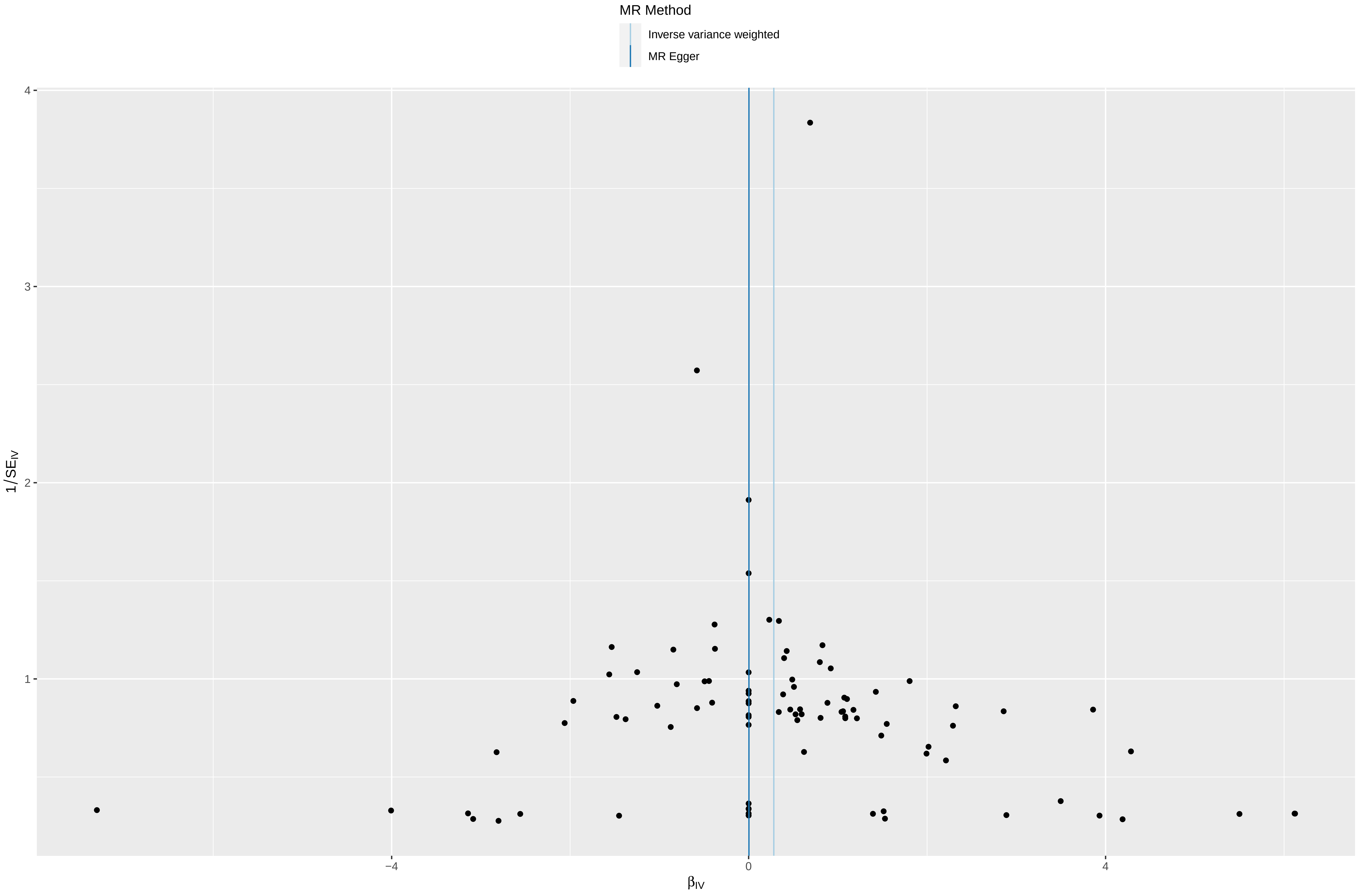

Supplementary figure 19 Forest plot of alcohol intake frequency and osteoarthritis risk

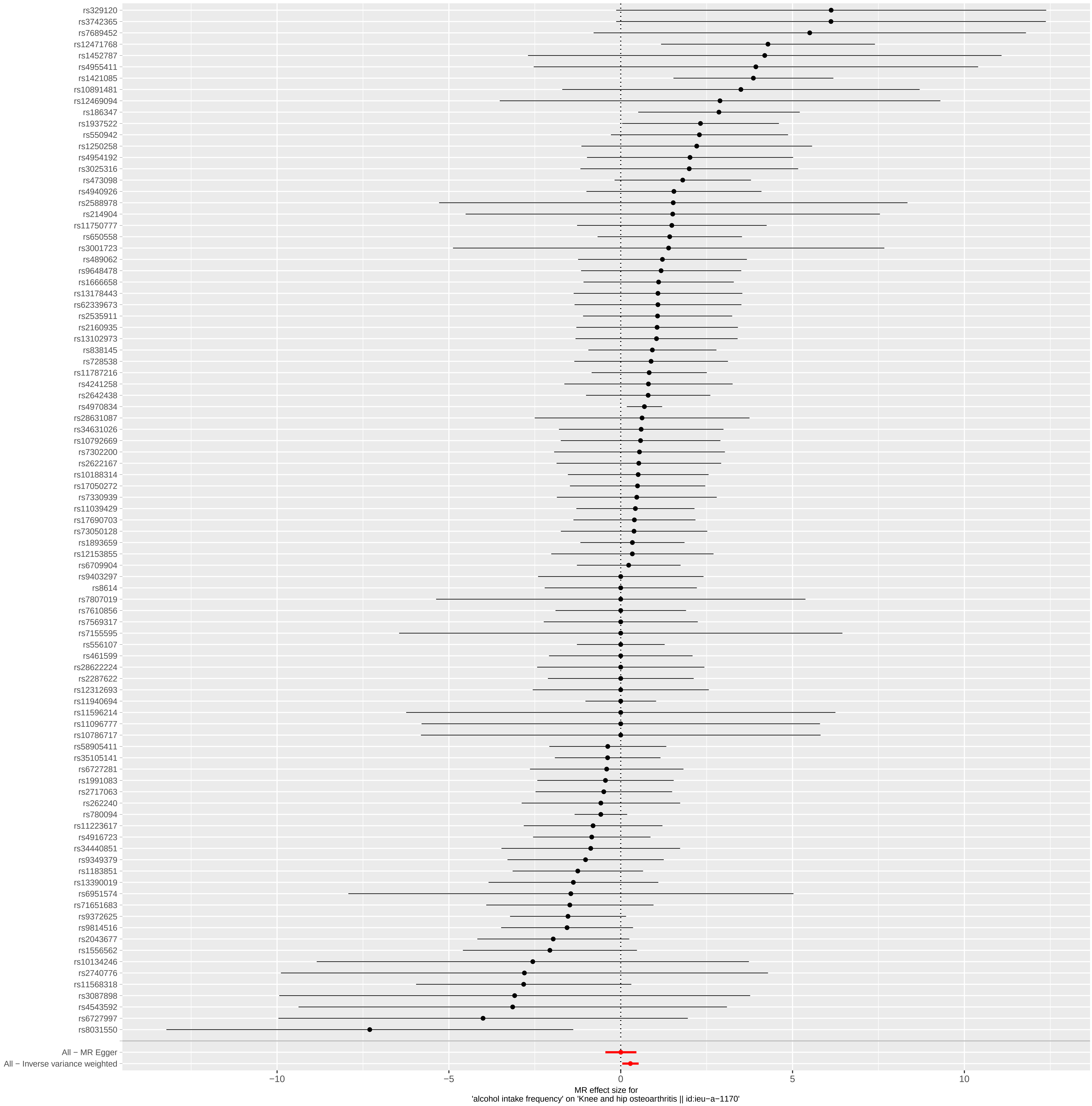

Supplementary figure 20 Sensitivity analysis of the "leave-one-out" method for alcohol intake frequency and osteoarthritis risk

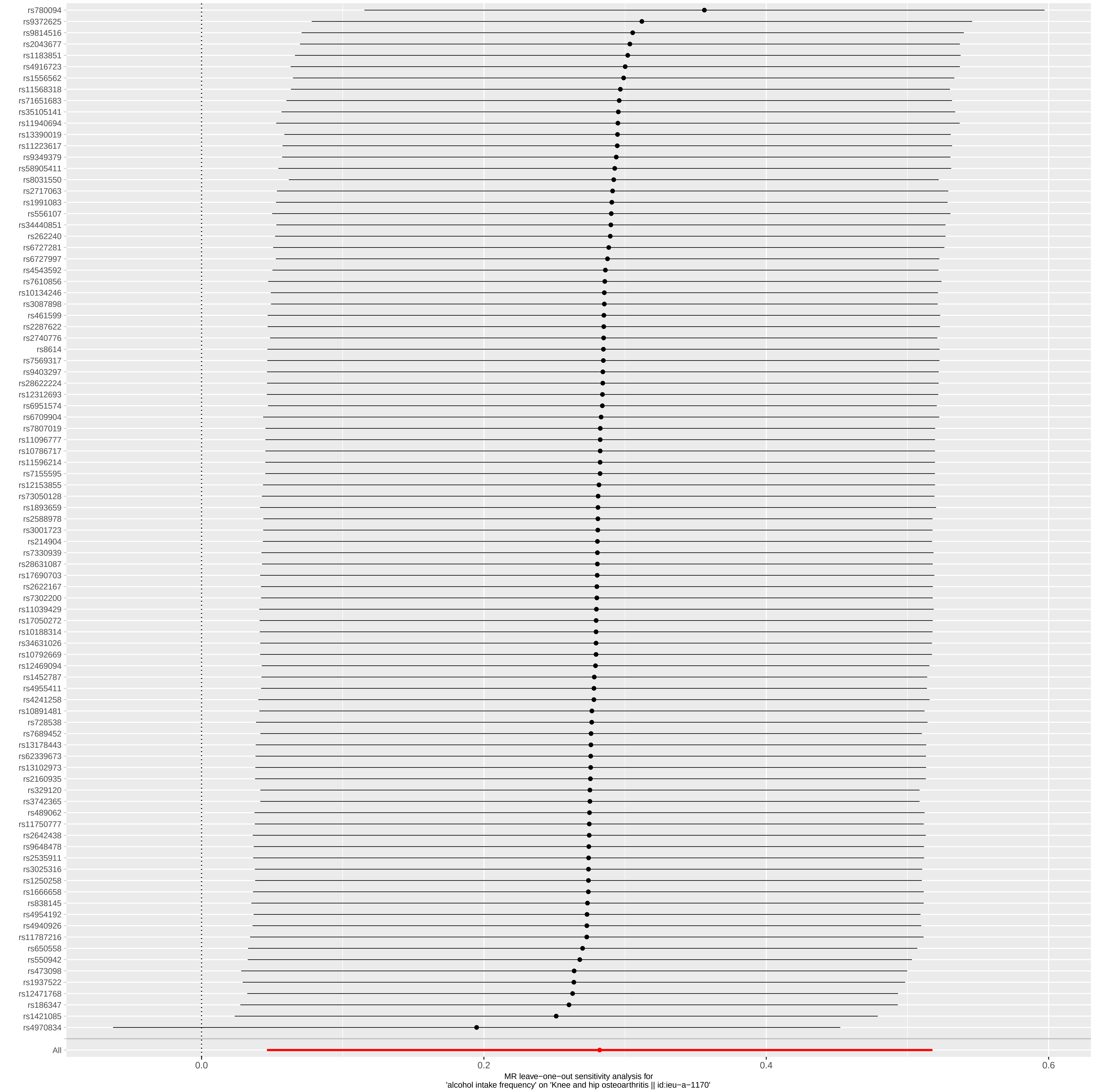

Supplement: Supplementary file 1 — Supplementary Material 1 [file 12889_2023_16250_MOESM1_ESM.zip › Supplementary materials WW/Supplementary materials/MR results visualization chats.pdf]
